# An established abdominal wall multidisciplinary team improves patient care and aids surgical decision making with complex ventral hernia patients

**DOI:** 10.1308/rcsann.2022.0167

**Published:** 2023-03-16

**Authors:** SG Parker, H Blake, S Zhao, J van Dellen, S Mohamed, W Albadry, H Akhtar, B Franczak, R Jakkalasaibaba, A Rothnie, R Thomas

**Affiliations:** ^1^ Croydon Health Services NHS Trust, UK; ^2^ St George’s University Hospitals NHS Foundation Trust, UK

**Keywords:** Abdominal wall, Abdominal hernia, Ventral hernia, Interdisciplinary communication, Patient care team

## Abstract

**Introduction:**

Abdominal wall reconstruction (AWR) is an emerging subspecialty within general surgery. The practice of multidisciplinary team (MDT) meetings to aid decision making and improve patient care has been demonstrated, with widespread acceptance. This study presents our initial experience of over 150 cases of complex hernia patients discussed in a newly established MDT setting.

**Methods:**

From February 2020 to July 2022 (30-month period), abdominal wall MDTs were held bimonthly. Key stakeholders included upper and lower gastrointestinal surgeons, a gastrointestinal specialist radiologist, a plastic surgeon, a high-risk anaesthetist and two junior doctors integrated into the AWR clinical team. Meetings were held online, where patient history, past medical and surgical history, hernia characteristics and up-to-date computed tomography scans were discussed.

**Results:**

Some 156 patients were discussed over 18 meetings within the above period. Ninety-five (61%) patients were recommended for surgery, and 61 (39%) patients were recommended for conservative management or referred elsewhere. Seventy-eight (82%) patients were directly waitlisted, whereas seventeen (18%) required preoperative optimisation: three (18%) for smoking cessation, eleven (65%) for weight-loss management and three (18%) for specialist diabetic assessment and management. In total, 92 (59%) patients (including operative and nonoperative management) have been discharged to primary care.

**Discussion:**

A multidisciplinary forum for complex abdominal wall patients is a safe process that facilitates decision making, promotes education and improves patient care. As the AWR subspecialty evolves, our view is that the “complex hernia MDT” will become commonplace. We present our experience and share advice for others planning to establish an AWR centre.

## Introduction

The prevalence of ventral hernia disease is rising due to an increasingly obese and ageing population.^1,2^ Over the past two decades there has been an increase in both clinical and academic activity attempting to lower postoperative hernia recurrence rates and reduce the burden of ventral hernia disease. To achieve this, abdominal wall reconstruction (AWR) is emerging as a subspecialty within general surgery, with increasing calls for specialised centres and specialised hernia surgeons.^3,4^ Indeed, in the UK, a group of abdominal wall surgeons under the auspices of the British Hernia Society published a ventral hernia triage algorithm in 2018 to facilitate sub-specialisation.^5^ More recently, a group of internationally renowned European abdominal wall surgeons published a landmark paper that outlines requirements and recommendations needed to establish an accredited abdominal wall centre.^6^ One of the many published criteria was for “A pre-operative multidisciplinary assessment of complex cases”, which was deemed as “*mandatory*”. In this publication, we outline the steps taken at Croydon University Hospital to establish an abdominal wall multidisciplinary team (MDT) meeting and report how this aids surgical decision making and improves patient care and outcomes.

## Methods

This is a prospective single-centre study, reporting a series of complex abdominal wall patients discussed in our abdominal wall MDT. Ethical permission for the study was obtained from our hospital’s research and development department. Data were collected from February 2020 to July 2022 inclusive. All consultants and trainees in our general surgery department were invited to attend our meetings and to refer patients for discussion. Additional specialists were invited to attend on an ad hoc basis. In accordance with current recommendations,^6–8^ the meetings were consistently attended by an upper gastrointestinal (GI) surgeon (and abdominal wall lead), two lower GI surgeons, a GI radiologist and a plastic surgeon. All other members of the department were strongly encouraged to attend and submit cases for discussion. Our anaesthetists and chronic pain specialists were invited to attend if their expertise was required. A tertiary bariatrics centre was available for referral of morbidly obese patients; however, owing to delays in local weight-loss service provision, patients were encouraged to lose weight when required. In recent meetings, surgeons from several other hospitals have attended virtually and have been invited to discuss their own challenging cases as well as contribute their views towards managing our own patients.

To acquire a detailed understanding, multiple datapoints were gathered for each case. All data were collected using a password-protected Excel spreadsheet (Microsoft Excel for Mac 2020, Version 16, Microsoft Corporation, Washington, US) and stored on a secure network server. Consequently, we collected each patient’s gender, age, body mass index (BMI) and smoking status. For hernia characteristics we recorded hernia type (primary, incisional, recurrent or parastomal), past surgical history, presence of a stoma or fistula, presence of a previous mesh (as well as mesh type and location) and formation of previous laparostomy. We collected the number of hernia defects and maximum width of the hernia defects. To assess morbidity, past medical history was collected with a particular focus on diabetes (latest glycated haemoglobin), cardiovascular disease and chronic obstructive pulmonary disease (COPD) because these have been shown to predispose to hernia recurrence.^9^ Anticoagulant prescriptions were also recorded. Perioperative risk assessment was scored for each patient using the American Society of Anaesthesiologists (ASA) score,^10^ the Portsmouth Physiological and Operative Severity Score for the Enumeration of Mortality and Morbidity (P-POSSUM)^11^ and the Carolinas Equation for Determining Associated Risks (CEDAR) score for postoperative wound events.^12^

The primary outcome of our MDT discussions was whether or not a patient was a candidate for surgical repair. If patients were recommended for an operation, we recorded whether preoperative Botulinum A toxin (Botox) was recommended, whether a component separation should be performed and if so whether an anterior component separation or a transversus abdominis release/posterior component separation was recommended. We recorded whether a mesh should be used and if so which and where it should be placed. We defined mesh location using the International Classification of Abdominal Wall Planes.^13^ Preoperative referral to a high-risk anaesthetic clinic, or to another specialty was documented. Where possible, anaesthetists with an interest in high-risk anaesthesia reviewed cases prior to attending the meeting. Patients considered inappropriate or unfit for surgery were reviewed in clinic and discharged with advice on conservative management. Postoperative patient outcomes were recorded prospectively, including systemic complications, local wound events, hernia recurrence, length of stay in the intensive care unit (ICU) and length of follow-up. To assess whether the AWR MDT aids surgical decision making we collected information on whether the patient had been discharged from the Croydon University Hospital general surgical department, irrespective of whether they had had an operation.

Our AWR MDT meetings were held every six to eight weeks using the Microsoft Teams business communications platform (Microsoft Corporation, Washington, USA). Prior to each meeting, patients' details were populated onto our abdominal wall proforma and uploaded onto Croydon University Hospital’s electronic patient record system. Consequently, each patient’s details and clinical histories were readily available for each meeting.

## Results

### Patient demographics and morbidity

Eighteen meetings were held over a 30-month period. In total 156 patients were discussed. Demographics and patient comorbidities can be found in Table 1. Ninety-eight (63%) patients were female and the median age of all patients was 58 years (26–86). Mean (sd) BMI was 34kg/mm^2^ (±7.8). Twenty-five (16%) patients were current smokers, 13 (8%) were ex-smokers (who had quit tobacco use more than 1 month prior to surgery) and 118 (76%) were non-smokers. Thirty-six (23%) patients had no comorbidities, 40 (26%) had one comorbidity and 80 (51%) presented with two or more comorbidities. Previous abdominal surgery had been performed on 138 (88%) of our patients. With regards to comorbidities, 27 (17%) had diabetes, 32 (21%) had a history of cardiac disease, 19 (12%) had COPD and 24 (15%) were on anticoagulants. Preoperative risk scoring revealed a mean (sd) ASA score of 2.46 (±0.75), a mean (sd) P-POSSUM score for morbidity of 23.9% (±19.4, range 0.4% to 96%), a mean (sd) P-POSSUM for mortality of 1.7% (±3.6, range 0.01% to 28%), and a mean CEDAR score of 34.4% (±23.2, range 3% to 90%).

**Table 1:** Patient demographics and comorbidities

Patient demographic/morbidity	Frequency
Female (%)	98 (63)
Median Age (range)	58 (26–86)
Mean BMI (sd)	34 (7.8)
**Smoking status (%)**	
Current	25 (16)
Ex-smoker	13 (8)
Non-smoker	118 (76)
**Comorbidity (%)**	
None	36 (23)
1	40 (26)
≥2	80 (51)
Previous abdominal surgery (%)	138 (88)
Diabetic (%)	27 (17)
Cardiac disease (%)	32 (21)
COPD (%)	19 (12)
On anticoagulants (%)	24 (15)
Mean ASA score (sd)	2.46 (0.75)
Mean P-POSSUM morbidity (sd)	23.9 (19.4)
Mean P-POSSUM mortality (sd)	1.7 (3.6)
Mean CEDAR score (sd)	34.4 (23.2)

ASA = American Society of Anaesthesiologists; BMI = body mass index; CEDAR = Carolinas Equation for Determining Associated Risks; COPD = chronic obstructive pulmonary disease; P-POSSUM = Portsmouth Physiological and Operative Severity Score for the Enumeration of Mortality and Morbidity

### Hernia characteristics

Table 2 describes the patients’ hernia characteristics. Thirty-one (20%) patients had a primary ventral hernia, 85 (54%) had an incisional hernia and 13 (8%) patients had a parastomal hernia. The remaining 27 (17%) patients presented with complex hernias: 7 (4%) had an inguinal hernia, 6 (4%) concurrent parastomal and incisional hernia, 6 (4%) concurrent divarication recti with primary umbilical hernia, 3 (2%) no hernia, 2 (1%) primary divarication recti, 1 (1%) spigelian, 1 (1%) concurrent incisional and inguinal hernia and 1 (1%) was a laparostomy healed by secondary intention. Forty-seven (30%) patients had previous mesh implants, 45 of these presented with recurrent hernia, 2 presented with chronic abdominal wall discomfort. Seven (54%) of the 13 parastomal hernias and 38 (45%) of the 85 incisional hernias were recurrent. The types of previous mesh implant are presented in Table 3. Three (2%) patients had no hernia defect, 106 (68%) had one defect, 24 (15%) had two defects and 23 (15%) had more than two defects. Mean (sd) defect width was 7.9cm (±8.04). Twenty-one (13%) patients had a stoma present at the time of surgery and 10 (6%) had a fistula.

**Table 2:** Hernia characteristics

Characteristic	Frequency
Primary ventral hernia	31
Incisional hernia	85
Parastomal hernia	13
**Complex hernias**	**27**
Inguinal hernia	7
Parastomal and incisional	6
Divarication recti with umbilical	6
Primary divarication recti	2
Spigelian	1
Incisional and inguinal hernia	1
Laparostomy	1
No hernia	3
**Recurrent hernias**	**45**
Recurrent parastomal	7
Recurrent incisional	38
**Hernia defects**	
0	3
1	106
2	24
>2	23
Mean defect width in cm (sd)	7.9 (8.04)
Stoma present (%)	21 (13)
Fistula (%)	10 (6)

**Table 3:** Frequency of previous implanted mesh type for recurrent hernia group

Previous mesh type	Frequency
Prolene	26
No record	11
Ventralex	5
Cellis	2
Ventralight	1
Strattice	1
Permacol	1

### MDT meeting outcomes

Of the 156 patients discussed, 95 (61%) were recommended for surgery as detailed in Table 4; 61 (39%) patients were advised against surgery. Seventy-eight (82%) of 95 patients were added straight to the waiting list, whereas 17 (18%) were asked to optimise modifiable risk factors prior to surgery: 3 (17%) were advised to stop smoking, 11 (65%) to lose weight and 3 (17%) to improve diabetic control. The reasons for advising against surgery were as follows: 18 (29%) patients had an excessively high risk for surgery, 15 (25%) cases were technically unfeasible, 10 (16%) patients had a tertiary referral for high-risk anaesthetic support, 8 (13%) patients had a plastic surgery referral at a different centre (prior to local involvement of a plastic surgeon at our centre), 6 (10%) were recommended to have further review in clinic and 4 (6%) were referrals to other specialities (2 [3%] pain team, 1 [2%] vascular and 1 [2%] bariatrics), see Table 4.

**Table 4:** Outcomes of multidisciplinary team discussion

Outcome (%)	Frequency
For surgery	95 (61)
Conservative management	61 (39)
**For surgery (%)**	
Added to waiting list	78 (82)
Optimise prior to surgery	17 (18)
Stop smoking	3 (17)
Weight loss	11 (65)
Diabetic control	3 (17)
**Conservative management (%)**	
Too high risk	18 (29)
No operation possible	15 (25)
Referred elsewhere for high-risk anaesthetic support	10 (16)
Referred to plastics	8 (13)
Review in clinic	6 (10)
**Referred to another specialty (%)**	**4 (6)**
Pain team	2 (3)
Vascular	1 (2)
Bariatrics	1 (2)
**Proposed procedures (%)**	
Preoperative Botulinum A	34 (36)
Retrorectus repair	30 (31)
Retrorectus repair +/− TAR	22 (23)
Retrorectus repair +/− ACS	8 (8)
Open inguinal repair	8 (8)
IPOM	7 (7)
Hernia repair (preperitoneal mesh) and abdominoplasty	4 (4)
Endoscopic component separation	2 (2)
Peritoneal flap repair	2 (2)
Inter-oblique flap repair	2 (2)
Simple suture repair	2 (2)
Complex surgery/multiple procedures	8 (8)

ACS = anterior component separation; IPOM = intraperitoneal onlay mesh repair; TAR = transversus abdominis release

Of the 95 (61%) patients put forward for surgery, preoperative Botox injections were recommended for 34 (36%). The proposed procedures are described in Table 4; 30 (31%) patients were advised to have a retrorectus mesh repair without adjuncts, 22 (23%) a retrorectus repair with either unilateral or bilateral transversus abdominis release, 8 (8%) a unilateral or bilateral anterior component separation with retrorectus mesh repair, 8 (8%) an open inguinal hernia repair, 7 (7%) an intraperitoneal onlay mesh repair and 4 (4%) a simple open hernia repair with preperitoneal mesh and abdominoplasty. A combination of endoscopic component separation, peritoneal flap repair, inter-oblique flap repair and simple suture repair were all advised for two (2%) patients, respectively. Eight (8%) patients were advised to have major complex surgery involving multiple procedures, including one (1%) laparostomy. Mesh type and location were also recommended. A summary of the MDT recommendations for mesh type and location can be found in Table 5.

**Table 5:** Proposed mesh type and location based on multidisciplinary team outcomes

Location of mesh/Type (%)	Frequency
Retrorectus prolene	38 (40)
Retromuscular prolene (incisional)*	9 (10)
Retromuscular cellis (parastomal)*	13 (14)
**Intraperitoneal/IPOM**	
Cousin biotech	1 (1)
Ventralight	3 (3)
Ventralex	1 (1)
Inter-oblique/peritoneal flap sandwich prolene	4 (4)
Inguinal prolene	8 (8)
Intraoperative decision	18 (19)

IPOM = intraperitoneal onlay mesh repair

*Retromuscular: transversus abdominis release performed so mesh sits in the retrorectus and preperitoneal planes, defined as retromuscular by International Classification of Abdominal Wall Planes^13^

### Operative and nonoperative outcomes

To date, 51 patients have had their operation with a mean (sd) follow-up of 358 days (±250, range 5–1,012). Twenty-four patients stayed on ICU, with a mean (SD) stay of 1.7 days (±3.5, range 1–7) and one outlier of 75 days. During the follow-up period, we recorded 16 (31%) postoperative complications and 2 (4%) mortalities. Both mortalities were due to causes unrelated to the indexed operation and occurred during the follow-up period. Six (38%) patients developed seromas (two [12%] infected), three (19%) wound infections, two (12%) hernia recurrences and one (6%) wound dehiscence. There was one (6%) case of intraoperative cardiac arrest during anaesthetic induction, one (6%) postoperative chest sepsis, one (6%) postoperative ileus and one (6%) bowel obstruction treated conservatively. Four (25%) patients required emergency wound exploration in theatre: two (12%) infected seromas, one (6%) flap necrosis and one (6%) wound dehiscence. At the time of writing, 92 (59%) of 156 patients discussed in our MDT have been discharged from Croydon University Hospital’s general surgery department. Thirty-seven (73%) postoperative patients have been discharged to primary care after hospital follow-up. Fifty-five (90%) patients considered unsuitable for surgery locally have been discharged back to primary care or referred elsewhere. These outcomes are summarised in Table 6.

**Table 6:** Operative and nonoperative patient outcomes

Outcome	Frequency
No. of AWR procedures to date (%)	51 (54)
Mean hospital follow-up, days (sd)	358 (250)
No. patients on ICU	24
Mean stay of ICU, days (sd)	1.7 (3.5)
**Complications (%)**	**16 (31)**
Seromas	6 (38)
* *Infected seromas	2 (12)
Wound infections	3 (19)
Hernia recurrence	2 (12)
Wound dehiscence	1 (6)
Intraoperative cardiac arrest	1 (6)
Chest infection	1 (6)
Ileus	1 (6)
Bowel obstruction	1 (6)
Wound exploration in theatre	4 (25)
Mortality*	2
**Nonoperative outcomes (%)**	
Discharged to primary care either postsurgery or for conservative management	92 (59)
Operative patients discharged	37 (73)
Discharged patients for conservative management	55 (90)

AWR = abdominal wall reconstruction; ICU = intensive care unit

*Occurred during follow-up period. Cause of death unrelated to indexed operation

## Discussion

In this paper, we present our experience of managing a large series of complex abdominal wall patients discussed in our MDT. It is our belief that managing complex abdominal wall patients is increasingly challenging and a forum that allows clinicians to discuss their cases facilitates decision making, improves efficiency, optimises outcomes and reduces risk to both patients and clinicians. In our series, 92 of 156 patients were discharged from the general surgical department at Croydon University Hospital either with or without an operation. Consequently, we conclude that an MDT enables us to make the most critical decision, on whether or not to operate. Decisions can be difficult for clinicians regarding safe and appropriate care for patients requiring complex AWR and defaulting too readily to a conservative, non-interventional “watch and wait” approach without exploring definitive options may be seen. This approach has the potential to delay decision making and increase pre-existing morbidity associated with complex hernias while overburdening an already oversubscribed outpatients department. The appropriate discharge of patients to primary care if they are unsuitable for surgical intervention, following discussion in an AWR MDT, reduces unnecessary follow-up in secondary care, which will not alter their ultimate management. This, in turn, reduces cost and improves efficiency.

AWR is a rapidly evolving subspecialty. Over the past 10 years, many new techniques have been published^14–17^ that claim to improve operative outcomes. Keeping up with the continual changes in operative technique,^18^ mesh composition,^19^ patient-reported outcomes^20^ and perioperative care is demanding, and an MDT forum can create educational opportunities allowing clinicians to stay up-to-date with the latest developments. This forum also promotes prospective data collection, allowing for the ongoing assessment of both patient characteristics and postoperative outcomes, which promotes learning and an improvement in the quality of care.

During the past 2 years our AWR service has evolved. The AWR MDT started as a collaboration between general surgeons and gastrointestinal radiologists, but now includes a plastics surgeon (WA), a chronic pain specialist (RJ) and a high-risk anaesthetic consultant (RJ). Our preoperative Botox pathway has been established, in collaboration with our inpatient pharmacy. To date, our anaesthetic colleague (RJ) has administered preoperative Botox to 16 patients. Working with our orthotics department, we can now provide all our major AWR patients with postoperative abdominal wall binders. Although there is little current evidence that this improves operative outcomes,^21^ we believe this practice cannot be detrimental. To improve outcomes we have also adopted new operative reconstructive techniques such as the peritoneal flap repair,^22^ inter-oblique peritoneal flap repair,^23^ endoscopic component separation^24^ and transversus abdominis release for parastomal hernia repairs.^25^ In the future, we aim to expand our research activity, improve our perioperative care pathways with pre-habilitation and weight loss programmes. Importantly, expanding into this new subspecialty requires energy from individual clinicians. Complex hernia surgery is a burgeoning and challenging subspecialty in its own right, yet widespread acceptance of it as such is yet to be seen. Consequently, it is likely that administrative and non-medical support staff, including MDT co-ordinators and specialist nurses, are not yet a common feature of many units undertaking this work. Currently, the MDT at Croydon University Hospital is almost entirely clinician led. Our hope is that administrative support will start to be provided for this emerging subspecialty within the foreseeable future.

### Study limitations

Our current MDT programme is not without its limitations. First, our current prospective database does not collect contamination grade, regarded by some as a vital perioperative factor to affect outcomes. Going forward, we intend to incorporate both the Ventral Hernia Working Group grade and the Centre for Disease Control grade into our prospective database. Second, our programme has not developed a system whereby we can follow up our patients long term. This would enable us to understand how both our operative and nonoperative patients are progressing, and again allow us to improve the care we provide if late complications were to arise. Quality-of-life questionnaires^20,26^ could also be used to further improve our understanding of how surgical repair affects our patients. We also look forward to broadening our retinue of meshes, to create bespoke management plans for our complex patients and assess their effects on longer-term outcomes. This includes a new biosynthetic mesh^27^ and a hybrid mesh^28^ for clean-contaminated cases and parastomal hernia repairs, respectively. Furthermore, our regional weight-loss and bariatric surgical service is based at a nearby tertiary hospital. Ideally, we would refer obese patients for surgery with the view of repairing their hernia after their weight-loss procedure. However, our experience is that waiting times for completion of a formal weight-loss process is time consuming with the result that the hernias themselves increase overall morbidity during their time awaiting metabolic surgery. Coordinating our MDT with the weight-loss pathway and coordinating in-house follow-up for this length of time is a considerable challenge. We therefore encourage patients to lose weight themselves, which often seems unachievable. To improve our service, we need to establish an abdominal wall patient pathway with our local bariatrics unit.

In conclusion, our AWR MDT has evolved over the past two years to become an integral part of the care we provide our patients. These are challenging cases that require collaboration between clinicians with discussions aimed at “trouble-shooting” and “problem-solving”. Consequently, our MDT platform prevents high-risk independent practice that could result in reckless surgery with poor postoperative outcomes. Consultants at Croydon University Hospital have found these meetings to be “helpful”, “informative”, “supportive” and “aid decision making, particularly on whether or not to operate”. For future work, we plan to improve our care by developing “the Croydon hernia bundle”. This “bundle” will provide preoperative and postoperative protocols designed to optimise patients prior to their surgery, hasten their discharge after surgery and improve long-term outcomes. These protocols will be evidence-based and we will continue to collect prospective data to see how they affect our outcomes.
